# Researching the delivery of health and nutrition interventions for women and children in the context of armed conflict: lessons on research challenges and strategies from BRANCH Consortium case studies of Somalia, Mali, Pakistan and Afghanistan

**DOI:** 10.1186/s13031-020-00315-8

**Published:** 2020-10-20

**Authors:** Michelle F. Gaffey, Anushka Ataullahjan, Jai K. Das, Shafiq Mirzazada, Moctar Tounkara, Abdirisak A. Dalmar, Zulfiqar A. Bhutta

**Affiliations:** 1 Centre for Global Child Health, The Hospital for Sick Children, 686 Bay St, Toronto, ON M5G 0A4 Canada; 2 Aga Khan University, Karachi, Pakistan; 3 Aga Khan University, Kabul, Afghanistan; 4 University of Sciences, Techniques and Technology of Bamako, Bamako, Mali; 5 Somali Disaster Resilience Institute, Mogadishu, Somalia

**Keywords:** Maternal and child health, Humanitarian health, Mixed methods, Research challenges, Somalia, Mali, Pakistan, Afghanistan

## Abstract

**Background:**

The BRANCH Consortium recently conducted 10 mixed-methods case studies to investigate the provision of health and nutrition interventions for women and children in conflict-affected countries, aiming to better understand the dominant influences on humanitarian health actors’ programmatic decision-making and how such actors surmount intervention delivery barriers. In this paper, the research challenges encountered and the mitigating strategies employed by the case study investigators in four of the BRANCH case study contexts are discussed: Somalia, Mali, Pakistan and Afghanistan.

**Discussion:**

Many of the encountered research challenges were anticipated, with investigators adopting mitigation strategies in advance or early on, but others were unexpected, with implications for how studies were ultimately conducted and how well the original study aims were met. Insecurity was a fundamental challenge in all study contexts, with restricted geographical access and concerns for personal safety affecting sampling and data collection plans, and requiring reliance on digital communications, remote study management, and off-site team meetings wherever possible. The need to navigate complex local sociopolitical contexts required maximum reliance on local partners’ knowledge, expertise and networks, and this was facilitated by early engagement with a wide range of local study stakeholders. Severe lack of reliable quantitative data on intervention coverage affected the extent to which information from different sources could be triangulated or integrated to inform an understanding of the influences on humanitarian actors’ decision-making.

**Conclusion:**

Strong local partners are essential to the success of any project, contributing not only technical and methodological capacity but also the insight needed to truly understand and interpret local dynamics for the wider study team and to navigate those dynamics to ensure study rigour and relevance. Maintaining realistic expectations of data that are typically available in conflict settings is also essential, while pushing for more resources and further methodological innovation to improve data collection in such settings. Finally, successful health research in the complex, dynamic and unpredictable contexts of conflict settings requires flexibility and adaptability of researchers, as well as sponsors and donors.

## Background

### Humanitarian context

The effects of war and armed conflict on the health and well-being of women and children are extensive, arising from direct exposure to violence, through forced displacement that intensifies social vulnerabilites, as a consequence of the destruction and disruption of health and other infrastructure and services, and through multiple other pathways [1, 2]. Recent estimates suggest that about 368 million children under 18 years old, 16% of the world’s child population, live in areas affected by armed conflict [3]. By the end of 2018, children accounted for only one third of the world’s population but 52% of its 25.9 million refugees [4] and about 40% of its 41.3 million internally displaced people [5]. The instrumental use of sexual violence, directed mainly but not exclusively at women and girls, is a common feature of many armed conflicts, with devastating physical, mental and social consequences [6, 7].

Given the extent of the effects of armed conflict on women and children, and the limited programmatic attention that these effects have received [8–10], the BRANCH Consortium (Bridging Research & Action in Conflict Settings for the Health of Women & Children) of international academic investigators and their research, implementation and advocacy partners have been working to improve the evidence base to support effective action on women’s and children’s health and nutrition in conflict settings through a set of interrelated workstreams using a range of methodologies and approaches [11]. These include efforts to better quantify the indirect effects of conflict on maternal and child mortality, and to synthesize and highlight gaps in the existing evidence and guidance on effective intervention delivery strategies in conflict settings. Another workstream has been the development and conduct of mixed-methods case studies to research the provision of sexual, reproductive, maternal, newborn, child, and adolescent health and nutrition (SRMNCAH&N) interventions in 10 conflict-affected countries: Pakistan, Afghanistan, Syria, Yemen, Somalia, South Sudan, Democratic Republic of the Congo, Mali, Nigeria and Colombia [12]. The aim of these case studies was to better understand the factors that have influenced the humanitarian health response for conflict-affected women and children in specific settings, including whether and how interventions for women and children have been prioritized and delivered. A mixed-methods case study approach was identified as the best means by which to obtain a comprehensive understanding of the complex contexts within which health services have been delivered in conflict settings, making use of both qualitative and quantitave data. This methodology allowed for an in-depth inquiry into the contextual factors that influenced humanitarian health actors’ programmatic decision-making and, in those cases where sufficient data were available, how those factors related to trends over time in service provision and intervention coverage.

The BRANCH co-investigators applied a number of criteria when selecting the specific conflict situations to study, aiming for a set of cases with wide geographical representation; a mix of acute, chronic or protracted, and recovery phases; a mix of displacement scenarios, including internally displaced populations and refugees in camps and open settings; and a mix of low- and middle-income settings. Additional considerations were the feasibility of conducting research in each candidate case setting with respect to security and access, the availability of local research partners, and the availability of existing data. A common protocol was developed including sample interview guides for collecting qualitative data from study participants and the specification of latent content analysis as the proposed qualitative analytical approach, as well as a master list of SRMNCAH&N interventions for which existing quantitative data (e.g., from national household surveys or health management information systems) should be sought to estimate time trends in coverage in each case setting. The range of humanitarian health actors to be targeted for recruitment included representatives or staff of UN agencies and international and local NGOs, ministry of health or other government officials, and health service providers. This common protocol was then adapted by individual case study research teams to ensure local relevance, acceptability and feasibility.

### Research studies

Detailed reports of the BRANCH case studies [13–22] as well as a cross-case synthesis of findings [23] are published elsewhere. For this additional paper, we aimed to highlight and discuss some of the research challenges faced and the corresponding mitigation strategies used by the international research teams conducting BRANCH case studies in Somalia, Mali, Pakistan and Afghanistan (Table 1). The Somalia case study was led by a team of researchers from the Somali Disaster Resilience Institute and focused on SRMNCAH&N intervention delivery since 2000 in the south-central Bay region and the capital Mogadishu, two areas heavily affected by the violence and armed conflict that the country has experienced since the start of civil war in 1991. The Mali case study was led by a team of researchers from the School of Public Health at the Faculty of Medicine and Odontostomatology at the University of Sciences, Techniques and Technology of Bamako, and focused primarily on SRMNCAH&N intervention delivery in the Mopti region since 2012, when widespread conflict erupted in northern Mali. Researchers from Aga Khan University led the Pakistan case study, focusing on SRMNCAH&N intervention delivery in the Makran division of Balochistan province, experiencing protracted conflict since 2005 between the government of Pakistan and local inhabitants over greater regional autonomy, and in the Federally Administered Tribal Areas (FATA) in the northwest (now part of Khyber Pakhtunkhwa province), an area of ongoing violence and insecurity since the arrival of Al-Qaeda and other foreign terrorists after the 2001 US invasion of Afghanistan and subsequent government military operations. The Afghanistan case study was also conducted by researchers from Aga Khan University, including those based in Kabul, and focused on SRMNCAH&N intervention delivery throughout the country since the 2001 US invasion, the most recent period in a decades-long experience of conflict and instability. Of the 10 case studies conducted overall, the four considered here were undertaken in collaboration with researchers from the Centre for Global Child Health at the Hospital for Sick Children in Toronto, which currently coordinates the BRANCH Consortium.

**Table 1:** Case country study areas, time periods, and local research leads

Country	Study area(s)	Study period	Local research lead
Somalia	Bay region; Mogadishu	2000–2018	Somali Disaster Resilience Institute (SDRI)
Mali	Mopti region	2012–2018	Faculty of Medicine and Odontostomatology, University of Sciences, Techniques and Technology of Bamako
Pakistan	Makran division, Balochistan province; Federally Administered Tribal Areas (FATA)	2006–2018	Aga Khan University, Karachi
Afghanistan	Nationwide	2001–2018	Aga Khan University, Karachi and Kabul

## Discussion

### Scientific importance of this research

Multiple sources of normative guidance on health intervention programming for women and children in humanitarian crises exist, but such guidance is not always evidence-based [24, 25]. This is, at least in part, because the evidence on intervention effectiveness in such settings is still limited [26–28]. Moreover, the existing relevant guidance rarely accounts for the specific, complex circumstances of providing health interventions and services in the context of armed conflict [29]. In order to further improve the humanitarian health response for women and children, it is imperative to better understand what the dominant influences on humanitarian health actors’ programmatic decision-making for women and children have been in recent and ongoing conflict situations, and how such actors have navigated the specific challenges that can impede effective delivery of interventions for women and children in those situations. Given the lack of sufficiently contextualized, evidence-based normative guidance for conflict settings, an improved understanding of prevailing decision-making processes will help identify and prioritize those areas in which guidance is most urgently needed. The BRANCH Consortium aims to support the development of such guidance, within WHO's existing mandate and framework, and in collaboration with other actors and networks dedicated to filling such guidance gaps. Moreover, the BRANCH Consortium will facilitate a series of regional workshops in 2021 to convene local and international humanitarian NGOs, civil society, UN partners and academics to discuss the specific regional and country-level implications of the Consortium’s case studies and other workstream findings and how they can inform ongoing and future humanitarian health action for women and children in specific conflict settings.

### Research challenges and mitigation strategies

A number of methodological challenges were encountered in multiple case study contexts. Many of these challenges were anticipated, with investigators adopting mitigation strategies in advance of protocol finalization or early on in the data collection process, but others were unexpected, with implications for how studies were ultimately conducted and how well the original study aims could be met. Here we discuss challenges posed by insecurity, complex sociopolitical contexts, and limited data availability. We also reflect on the difficulties of maintaining methodological consistency across the case studies.

### Insecurity

The personal safety of study personnel and participants are critical considerations for research planning in the context of armed conflict, given the attendant risks to conducting or participating in study activities in insecure settings, including potential exposure to armed violence, abduction, intimidation and other threats. Almost inevitably, such risks then impose logistical, behavioural and other constraints on study personnel and participants. Predictably, insecurity was a fundamental research challenge in each of the four BRANCH case studies discussed here.

In all four cases, physical access to some of the targeted study geographies, and thus the potential study participants, was constricted because of insecurity in or en route to those geographies. In Pakistan, some areas of study interest in the northwest border region were deemed ‘notified’ areas by the government, meaning local residents had been notified that military operations would be conducted and that they should leave the area [30]. Until such areas are de-notified by the military, special permission to access and work in those areas is needed and is difficult to obtain. Recruited study participants from such areas had to be brought out to the provincial capital for interviews or were interviewed remotely. Destruction of infrastructure and disordered road networks and communication systems as a consequence of armed conflict further hindered access to potential and actual study participants in both Afghanistan and Pakistan, with challenging terrains in focal areas in both countries further curtailing access. At the time that the Somalia case study was conducted, most international agencies and organizations focusing on health and development in Somalia, as well as many Somali ones, managed operations remotely, either out of Nairobi or from within the secure compound at the international airport in Mogadishu. Fortunately, the local research team was able to conduct face-to-face in-depth interviews with many study participants not only in Nairobi but also in Mogadishu and Baidoa. However, there were still some study participants located in geographies that the study team could not access, and those interviews were therefore conducted remotely by videoconference. In Mali, persistent insecurity in the study area of Mopti meant that local research staff had to minimize the time they physically spent in the field to collect data. When conditions were favourable, multiple data collection teams were deployed simultaneously to take maximum advantage of those limited windows of opportunity.

Limited access to the field because of security considerations also limited interaction between study team members, especially between local and non-local team members, with implications for the efficient provision of technical support and for effective and meaningful methodological capacity-building. At times, the necessary reliance on remote rather than in-person collaboration between study personnel caused delays in data collection and variability in data quality. For example, with limited opportunity for non-local and, at times, local co-investigators to accompany field staff while conducting in-person interviews, opportunities for real-time supervision and concurrent training were also limited; often, transcripts had to be generated and shared before it was apparent where further training of field staff on study objectives, probing techniques, or other areas was needed. To help compensate for limited in-person interaction, study teams in all four case studies employed several different modes of communication, including email and video calls as well as instant messaging through applications such as WhatsApp. The use of multiple communication modes helped ensure that feedback, queries and ideas were exchanged in as timely a manner as possible. Local and non-local teams also sought to meet in-person as frequently as possible, usually outside of the focal study area. Meetings of the local and non-local research personnel for the Somalia and Mali case studies took place in Nairobi and Bamako, respectively, for example, and in Karachi for the Pakistan and Afghanistan studies.

In addition to the personal safety risks associated with conducting fieldwork in insecure settings, researchers living or working in conflict zones may also be put at risk by the publication of their study reports or journal articles that explicitly or implicitly identify, characterize or assign culpability to specific actors involved in an ongoing conflict. This was a consideration when preparing the primary publication of the Somalia case study, for example.

Study participants also assumed some risk by engaging in these case studies. For some study participants, stated concerns related not only to their physical safety but also to their job security. Operating in conflict settings meant that some organizations had to negotiate with armed non-state actors in order to deliver services to vulnerable populations. Such negotiations are illegal in some countries and can result in organizations being blacklisted, and so this practice is not often openly discussed. In the context of the Somalia study, multiple interview participants alluded to negotiating with armed non-state actors but refused to provide further detail when probed, given the potential repercussions for themselves and their organizations. Fears about the potential repercussions of study involvement also influenced study participation in Afghanistan and Pakistan, where local study personnel found it difficult to secure the participation of some local NGOs given a prevailing mistrust of foreign NGOs and UN agencies in some of the focal study communities. Fortunately, repeated discussion and assurance of maintaining confidentiality ultimately secured the participation of most potential local NGO study subjects. However, for some interview questions, some participants did request the interviewers to suspend the audio recordings.

### Navigating local sociopolitical contexts

International collaborative research, in any setting, often relies heavily on local research partners’ knowledge and networks that provide insight into the local sociopolitical context and, usually, an advantage in navigating that context. This may be especially true in conflict settings, where sociopolitical realities are arguably more complex than in other humanitarian or development settings. To meet the studies’ aim of better understanding the influences on the humanitarian health response for women and children in specific conflict settings, local research teams were key to identifying the most appropriate focal study areas, the range of stakeholders from whom it would be essential to get buy-in, the most appropriate participants to target for recruitment and how best to access them, and other study parameters. Local research partners also ensured that the study methods and tools were appropriate for the cultural context. In Somalia, for example, interview questions about the delivery of family planning services were modified to refer to the promotion of child spacing, and female study participants were interviewed exclusively by female study personnel. In Afghanistan and Pakistan, local partners advised the avoidance of interview questions about local NGO communication or coordination with opposition forces or anti-state elements to facilitate intervention delivery, or questions about the role of security agencies.

However, navigation and negotiation still proved difficult for local study personnel in some cases, particularly with respect to securing the participation of international NGOs in the study, either as interview participants or as sources of documents or data. In both the Somalia and Mali cases, the non-local co-investigators additionally approached their own global or regional headquarter-level NGO and UN agency contacts about the studies, requesting them to further sensitize their country-level counterparts and encourage their participation. This approach helped to promote broad stakeholder representation at the study inception meetings that were held early on in the research planning cycle, where representatives of government ministries and humanitarian actors including NGOs and UN agencies were invited to attend. At each meeting, the research team presented the proposed case study plan; sought feedback from attendees on the proposed geographical focus, topics of inquiry and other methodological considerations; and solicited input on potentially relevant sources of quantitative data and potential interview participants. The input from meeting attendees was essential in finalizing the study-specific protocols. In Somalia and Mali, leveraging existing relationships between international research personnel and HQ-level NGO and UN personnel helped to facilitate and ensure the utility of the study inception meetings, and in some cases also expedited permissions for the sharing of quantitative data; it did not ultimately improve interview participation rates, however. While many stakeholder representatives attended and provided valuable feedback at the study inception meetings, several still refused to be interviewed individually as study participants, even when explicitly permitted or otherwise encouraged by their organizational headquarters.

In Mali, in addition to recruiting representatives from government and the public sector, NGOs, and UN agencies as qualitative interview participants, the local research team was also prepared to recruit and interview study participants from among the ranks of local rebel groups. For the wider research team however, including international co-investigators, a sufficiently robust risk assessment couldn’t be made that would warrant team-wide support for this approach, and so representatives from rebel groups were ultimately not targeted for study recruitment. In Somalia, the illegality of negotiating with armed non-state actors did not allow for such an approach to even be considered.

### Lack of reliable intervention coverage and other quantitative data

Given that the case studies aimed to better understand the factors that have influenced the humanitarian health response for conflict-affected women and children in specific settings, including whether and how interventions for women and children have been prioritized and what the barriers to and facilitators of their delivery have been, the reconstruction of intervention coverage trends in the focal geographies and populations was a key objective of each mixed-methods study. However, the collection of high-quality data on the coverage of health and nutrition interventions is extremely challenging in conflict settings [25, 31, 32]. Organizations involved in humanitarian health response may conduct rapid surveys to assess morbidity and mortality, as well as health service availability and quality, but cannot always achieve representative samples. Large-scale national surveys such as Demographic and Health Surveys (DHS) or Multiple Indicator Cluster Surveys (MICS) measure intervention coverage in many areas reasonably well, but they may be unable to sample populations in highly insecure areas and usually have relatively long periods between survey rounds. Where health facility data exist and are available for analysis, these may be inaccurate and incomplete, with record-keeping being disrupted by conflict events and interrupted by facility closures, or potentially compromised by political or other influences. Moreover, dynamic population movement and mass displacement can render both population- and facility-based data, and the results of the analyses that are conducted with them, difficult to interpret. Such quantitative data were therefore used cautiously in the case studies, with study teams collating and analysing the available data, but critically and with explicit uncertainty.

In the Afghanistan and Pakistan case studies, additional analyses investigated trends in intervention coverage stratified by conflict intensity. The research teams used a novel Delphi approach to classify the intensity of conflict in each province in Afghanistan and in each district or agency in Balochistan or FATA in Pakistan by soliciting consensus opinion from a panel of experts in each country on the extent to which access to and provision of health services was affected by armed conflict in each area [17, 18]. This approach supplemented an assessment of conflict intensity based on the number of direct deaths resulting from conflict events in a given time and space (“battle-related deaths”), based on data from the widely used and publicly available database compiled by the Uppsala Conflict Data Program [33]. The extent to which expert consensus-based approaches to humanitarian health data collection could or should be adopted in other conflict settings with sparse or unreliable population-based data is unclear and could be an area of future methodological research.

### Maintaining consistency across studies

Across these four studies, and indeed across the set of all 10 BRANCH case studies, it was difficult to maintain fidelity to a common methodological approach. While an initial global protocol was developed as a reference for all case studies, each research team then derived a case-specific protocol based on local conditions and constraints. The studies ultimately differed in multiple ways therefore, including in their scope (in terms of time and space, for example, or the specific domains of women’s and children’s health on which they focused) and in terms of the data that could be collected and the analyses that could be undertaken. Mindful of this challenge from the outset, the BRANCH Consortium’s intention was not to produce wholly comparable case studies, but to be able to identify existing trends and synthesize common elements and important learnings across cases. Maintaining a common approach across multiple studies is difficult in any research setting but perhaps especially so in conflict settings, where the logistical and behavioural constraints imposed by insecurity, and by its unpredictability, demand flexibility and adaptability from research teams and from their sponsors and funders.

## Conclusions

A few key learnings emerge from this discussion of the research challenges faced and mitigation strategies used by the international research teams conducting BRANCH case studies on health and nutrition intervention prioritization and delivery for conflict-affected women and children in Somalia, Mali, Pakistan and Afghanistan. First, strong local research partners are essential. Such partners bring not only technical and methodological capacity to develop or adapt and then execute a study protocol, but also the acumen and insight that is needed to truly understand local dynamics, accurately interpret those dynamics for the wider study team, and effectively navigate those dynamics to ensure the rigour and relevance of the study. Second, researchers must have realistic expectations of existing data, and more particularly, the inferences that can reasonably be made from analyses undertaken with those data. It is useful and important to undertake such analyses, but they must be interpreted with caution, and the push for more resources and for further methodological innovation to improve data collection in the context of armed conflict must continue. Finally, successful health research in the complex, dynamic and unpredictable contexts of conflict settings requires researcher flexibility and adaptability, ideally supported by the same from research sponsors and donors.

## Data Availability

Not applicable.
